# Smoking Behaviour before, during, and after Pregnancy: The Effect of Breastfeeding

**DOI:** 10.1100/2012/154910

**Published:** 2012-03-12

**Authors:** Laura Lauria, Anna Lamberti, Michele Grandolfo

**Affiliations:** National Center of Epidemiology, Surveillance and Health Promotion, National Institute of Health, 00161 Rome, Italy

## Abstract

Data for this study were obtained from a population-based follow-up study in 25 Italian Local Health Units (LHUs) to evaluate pregnancy, delivery, and postpartum care in Italy. A sample of 3534 women was recruited and interviewed within a few days of their giving birth and at 3, 6, and 12 months after delivery, by trained interviewers using questionnaires. The objective of the study was to evaluate changes in smoking behaviour from one interview to the next. Of 2546 women who completed the follow-up, smoking prevalences before and during pregnancy were 21.6% and 6.7%; smoking prevalences and smoking relapse at 3, 6, and 12 months were 8.1% and 18.5%, 10.3% and 30.3%, and 10.9% and 32.3%, respectively. Smoking during and after pregnancy was more likely among women who were less educated, single, not attending antenatal classes, employed, and not breastfeeding. The results show that women who are breastfeeding smoke less than not breastfeeding women, even after controlling for other predictors (i.e.,  smoking relapse at 12 months: OR = 0.43, 95%  CI:  0.19, 0.94). A low maternal mood increases the risk of smoking relapse within 6 months of about 73%. This study also suggests that prolonged breastfeeding reduces the risk of smoking relapse and that this reduction may be persistent in time. Interventions targeting breastfeeding promotion may also indirectly support smoking cessation, even in absence of specific interventions.

## 1. Introduction

Smoking is one of the most important avoidable causes of disability, mortality, and adverse maternal and fetal outcomes in Western countries [1, 2].

During recent decades, researchers have studied the adverse effects of smoking on conception, pregnancy, fetal, and child health [3, 4]. The associated adverse outcomes include low birth weight, reduced fetal growth, placenta previa, preterm birth, respiratory infections, asthma, sudden infant death syndrome [3–6], and hyperkinetic disorders [7].

In recent years, almost all Western countries have registered a decrease in smoking prevalence in the general population and in particular among pregnant women. Data from the Pregnancy Risk Assessment Monitoring System (PRAMS) show a slight reduction in smoking prevalence during pregnancy, in the period 2000–2005, from 15.2% to 13.8% [2]. However, these values remain far from the goal of 2% during pregnancy given by “Healthy People—2010” [8]. After a decreasing trend, the prevalences of smoking in pregnancy were 11% in Canada in 2005 [9], 16% in Denmark in 2005 (among pregnant nulliparous women) [10], and 20% in Australia in 2004 [11]. An opposite trend was registered in Japan where the prevalence of smoking in pregnancy increased from 5.6% in 1991 to 10% in 2001 [12]. Moreover, an increasing trend has been forecast for Eastern Europe and Asiatic countries [2]. According to the last European report on perinatal health, more than 10% of women smoke during pregnancy with values ranging from 5% in Lituania to 22% in France [13].

In Italy, the prevalence of smoking during pregnancy declined from 9.2% in 2000 to 6.5% in 2005. The percentage of smokers who quit during pregnancy increased from 63.4% in 2000 to 70.7% in 2005; it has also been estimated that almost one third of quitters do so permanently [14]. Nevertheless, more recent surveys show that in 2009 there was an increase in the prevalence of smoking in the general population and specifically in women of reproductive age [15, 16].

Several studies have shown that pregnancy is a period when many smokers quit, although most of them relapse within one year of delivery. About 40% of smokers quit during pregnancy, but of them, 45% relapse within 3 months from delivery, 60–70% within 6 months, and almost 80% within one year [17]. In order to plan effective health promotion interventions, several studies have focused on the factors which predict quitting smoking during pregnancy and relapsing postpartum. Factors considered as potential predictors of the smoking behaviour change during and after pregnancy are socioeconomic status, education, stress, living with a smoker, marital status, age, parity, employment, and breastfeeding [2, 18]. Breastfeeding is one of the strongest factors associated with smoking abstinence after pregnancy [19–21]. Nevertheless, the nature of the relationship between breastfeeding and smoking needs further research. It is not clear if breastfeeding promotes smoking cessation or smoking cessation prolongs breastfeeding or, alternatively, if there is a third unidentified factor such as stress or family/social support, which may influence both breastfeeding and smoking behaviour [21–23]. In this study we investigated the effect of maternal mood and participation in antenatal classes (ACs).

The aims of this study are (i) to explore the prevalences of smoking before, during, and at 3, 6, and 12 months after pregnancy, (ii) to investigate the factors associated with smoking cessation during pregnancy and smoking relapse after pregnancy, and (iii) to focus on the relationship between breastfeeding and smoking relapse.

## 2. Materials and Methods

Data for this study were obtained from a population-based follow-up study of maternal behaviors and maternal health-care services during preconception, pregnancy, and postpartum conducted by the Italian Institute of Public Health in 2009 [24]. The survey was offered to all of the 20 Italian administrative Regions, but only 11 agreed to participate. Each region is divided into administrative Local Health Units (LHUs). Twenty-five of the 79 LHUs present in the participating Regions, participated in the study. Women who had given birth and resident in these LHUs were the target population (about 45000 new births each year). Both town and hospital registers of births were used as sampling frame.

A pseudo-random sampling method was used to recruit 120 women who had given birth in each LHU. All women who had given birth were recruited until 120 were reached in each LHU. Exclusion criteria were as follows: severe illness of mother or child; women with an active infection and fever >38°, and women with hematic loss >1000 cc.

In order to make the entire sample representative of the total population from which the LHU samples were derived, descriptive and multivariate analyses were weighted by the reciprocal of the sampling fractions. Women were interviewed face to face (91%) or by phone (9%) by trained interviewers using a questionnaire, preferably in hospital after delivery or within a few days from discharge. The reinterviews of the same women were conducted by phone after three, six, and twelve months from delivery. At the end of each interview, the women were asked to give their consent to be reinterviewed at the successive follow-up date. The first questionnaire was structured in four sections regarding pregnancy, delivery, postpartum, and sociodemographic characteristics. For multiparous women, items regarding breastfeeding of the previous children were also included. The follow-up questionnaires included items regarding extra hospital assistance, breastfeeding, smoking behaviour, and a set of items aimed at defining a general maternal mood. The basic characteristics of women lost to follow-up were compared with those of women who completed the follow-up by the Pearson *χ* ^2^ test. Smoking behaviour was the main outcome variable of this study. Prevalences of smoking were estimated as the ratio between the number of women who reported to be smoker at each point interview and the total number of interviewed women. Descriptive analyses on prevalence of smoking, on quitting smoking, and on smoking relapse within 3, 6, and 12 months from partum were reported. Weighted multivariate logistic models which take account of complex survey data were used to analyse the association between breastfeeding and smoking status after controlling for potential confounding factors. If not otherwise indicated, the analyses focused on women who completed the follow-up and were conducted using the statistical software STATA, version 11.

## 3. Definition of Variables

At delivery the women were asked two questions about their smoking behaviour: if they smoked regularly before pregnancy and if yes, if they also smoked during pregnancy. At 3, 6, and 12 months after delivery, the women were asked if they smoked regularly. Possible answers for all the questions on smoking behaviour were no or yes with the number of cigarettes per day specified. On the base of the number of cigarettes smoked before pregnancy, women were classified as light smokers (≤10 cigarettes/day) or heavy smokers (>10 cigarettes/day).

Two different definitions of breastfeeding were used: “full breastfeeding” when children were exclusively breastfed or breastfed with addition of water/herbal teas (no formula was used), and “any breastfeeding” for any other addition to maternal milk. “Full breastfeeding” was used to predict smoking relapse at 3-month follow-up; “any breastfeeding” was considered at the 6- and 12-month follow-ups. Education was classified as low (less than high school), medium (high school), and high (university). Participation in antenatal classes (yes, no), consisting of a variable number of meetings of women and maternal/child care professionals during the present pregnancy and in puerperium, was also investigated. Employment status at each follow-up time was classified as not employed, employed who had restarted working after pregnancy, and employed who had not yet restarted working. A measure of “maternal mood” at each follow-up interview was created by combining the answers to 7 questions which compared life conditions after delivery with those before conception. The first item required women to indicate: if their life was worse, improved, or not changed; the next four items required women to indicate if they felt more, less, or as before baby arrived with regard to (1) serenity, (2) tiredness, (3) whether they were “understood”, (4) whether they were “supported”, (5) the time they had for themselves, and (6) the attention paid to them. Women were considered in an uncomfortable status (low mood) if a worsening was indicated in 3 or more of the items. Maternal age at delivery was categorized in two classes, ≤30 years and >30 years. Marital status was categorized as married and unmarried at the time of delivery; the latter category included women who were single, divorced, separated or widowed. Parity was categorized as primiparous and multiparous. In a subgroup analysis, multiparous women were categorized as multiparous who breastfed the previous child for less than 3 months and multiparous who breastfed the previous child for more than 3 months.

## 4. Results

A total of 3669 women met the eligibility criteria; 135 women were declined or were not found. The 96.3% (3534) of eligible women were recruited and interviewed at delivery; 2546 (72%) were reinterviewed at 3, 6 and 12 months (Table 1). Women who completed the follow-up were not statistically different from women lost to follow-up with respect to the smoking behaviour before (21.6% versus 24.8%) and during pregnancy (31.3% versus 33.0%), complete breastfeeding after delivery (70.4% versus 67.5%), and marital status (77.6% versus 73.2% of married women); they were more likely to be primiparous (55.5% versus 49.8%), older (>35 years: 32.4% versus 28.2%), highly educated (23.8% versus 19.4%), employed (71.8% versus 63.7%), and participating in the AC (40.7% versus 23.7%).

The following analysis focuses on the women who completed the follow-up. Most of them were older than 29 years (72.0%), of medium/high education (72.6%), married (77.6%), employed (71.8%), primiparous (55.5%), not smokers (78.4%), and not partecipating in AC (59.3%).

The prevalence of smoking was 21.6% before and 6.7% during pregnancy. At 3-, 6-, and 12-month follow-up, prevalence (prevalence among breastfeeding) was 8.1% (5.2%), 10.3% (5.0%), and 10.9% (6.6%), respectively (data not reported in table).

In Table 2, the changes in smoking behaviour at different points in time, controlling for the number of smoked cigarettes before pregnancy, are reported. Among 580 smokers before pregnancy, 68.7% reported that they quit smoking during pregnancy. Light smokers were more likely than heavy smokers to quit smoking during pregnancy. Of the 183 (31.3%) smokers who reported that they continued to smoke during pregnancy, about 66% (113) reported that they reduced their consumption of cigarettes.

Among the 397 women quitting smoking during pregnancy, 18.5% (9.5% in case of “any breastfeeding”) relapsed within 3 months, 30.3% (14.3% in case of “any breastfeeding”) relapsed within 6 months, and 32.3% (21.1% in case of “any breastfeeding”) relapsed within 12 months. Percentages of smoking relapse appear higher for heavier smokers.

In Table 3, the results of the logistic regression models for smoking prevalence before pregnancy, quitting smoking during pregnancy, and relapse smoking at 12-month follow-up are reported. Tobacco use before pregnancy was more likely for unmarried with respect to married women (OR = 2.30, 95% CI: 1.75, 3.02). Protective factors were medium and high education with respect to low (medium OR = 0.65, 95% CI: 0.48, 0.89; high OR = 0.50, 95% CI: 0.37, 0.67). The same regression model was also used for a subgroup analysis of pluriparous women (*n* = 1619) who were categorised with respect to the type of breastfeeding of their previous child. In comparison with pluriparous who did not breastfeed or breastfed their previous child for less than three months, tobacco use was less likely for pluriparous women who have breastfed previous child for more than 3 months (OR = 0.58, 95% CI: 0.42, 0.80) (not reported in table).

To quit smoking during pregnancy was more likely for medium and highly educated women than less educated women (medium education OR = 1.76, 95% CI: 0.93, 3.32; high education OR = 2.42, 95% CI: 0.94, 6.27). Quitting was less likely for unmarried women (OR = 0.38, 95% CI: 0.21, 0.71), for heavy smokers (OR = 0.45, 95% CI: 0.31, 0.64), and for multiparous women (OR = 0.61, 95% CI: 0.39, 0.96).

Among the 397 women quitting smoking during pregnancy, restarting at 12 months of follow-up, was positively associated with having been a heavy smoker (OR = 2.38, 95% CI: 1.44, 3.91); protective factors were participation in AC (OR = 0.43, 95% CI: 0.19, 0.96) and any breastfeeding at follow-up time (OR = 0.43, 95% CI: 0.19, 0.94). Women who were employed before pregnancy, and especially women who had not yet restarted working at the follow-up time, were less likely to relapse compared with women not employed (restart working OR = 0.47, 95% CI: 0.21, 1.06; not restart working OR = 0.36, 95% CI: 0.09, 1.36).

Logistic regression models were also used to analyze smoking relapse within 3-, 6-, and 12-month follow-up considering all the women interviewed at each follow-up point without the restriction on women completing the follow-up at 12 months and including the potential predictor “maternal mood” (Table 4). The effect of breastfeeding was statistically significant in the all three points but higher at 3 and 6 months in comparison to 12 months. The variable “maternal mood” shows also a statistically significant effect on smoking relapse at 3 months (low mood OR = 1.73, 95% CI: 1.21, 2.47) and at 6 months (low mood OR = 1.73, 95% CI: 1.03, 2.90), but not at 12 months (low mood OR = 0.71, 95% CI: 0.46, 1.10). We explored a potential interaction effect between breastfeeding and maternal mood at each follow-up time but no statistically significant effect was found.

## 5. Discussion

This population-based survey gave us the opportunity to investigate the prevalences and the changes in smoking behaviour of women who have given birth, from before conception to 1 year after delivery. The prevalence of smoking before pregnancy (21.6%) was similar to that estimated by the Italian National Institute of Statistics (ISTAT) [14] in 2005 (20.1% for women aged 25–34 years and 21.7% for women aged 35–44 years). Analogously, similar values of smoking prevalence during pregnancy (6.7%) (ISTAT 6.5%) and percentage of smoking women who continued to smoke in pregnancy (31.3%) (ISTAT 29.3%) were also found. The closeness of these results may indicate that our sample is representative. Nevertheless, the percentage of women who were lost at follow-up (28%) are a limitation to the accuracy of the prevalence estimates after delivery. The respondents and nonrespondents were different with respect to socio-demographic characteristics, but not in smoking behaviour before and during pregnancy and in breastfeeding at birth. In addition, the multivariate analyses at 3- and 6-month follow-up were based on the total interviewed women at each follow-up point, 92% of the original sample at 3 months, and 91% at 6 months. Results did not differ if the multivariate analyses included only women who completed the follow-up, suggesting that those lost to follow-up may not introduce an important bias.

Another potential limitation of this study is that the measures of smoking status were self-reported and did not include biochemical validation of tobacco use. Studies have shown that self-reported measures of smoking status may be underestimated [25–27], and also, quitting smoking rates in pregnant women may be overestimated [28]. However, research has also shown that the accuracy of self-reported smoking status varies according to the settings in which the questions are asked. More specifically, interviewer-administered questionnaires, observational studies, reports by adults, and biochemical validation with cotinine plasma have been found to be associated with higher estimates of sensitivity and specificity [29]. Moreover, our previous study conducted with the same collection methods, on the association between smoking status and smoking intensity and low birth weight, showed results consistent with data reported in the literature, suggesting, indirectly, that the collected information was not strongly affected by these biases. Comparing our results with those of other Western countries, although the reference periods and the methodological approach may be different, smoking prevalences before pregnancy and during pregnancy are the lowest in Italy (21.6% and 6.7%, resp.) compared with the USA 22.4% and 14% [30, 31], Japan 29.3% and 10.0% [18, 32], France 35.9% and 21.8%, [13], and UK 33.0% (prevalence before or during pregnancy) and 17.0% [13]. Our prevalence results are also the lowest when compared with the sample-based estimates of other European countries: Belgium 22.4% and 16.0%, Bulgaria 33.2% and 7.0%, Germany 38.7% and 16.9%, Greece 30.0% and 11.7%, Ireland 66.5% and 52.5%, and Portugal 25.7% and 14.3%; [33]. The percentage decline in smoking prevalence during pregnancy was higher in Italy (68.5%) than in all the other countries except Bulgaria (79%). Nevertheless, tobacco use by pregnant women remains an important problem considering that the prevalence is far from the 2% goal in healthy people fixed in 2010 in USA [8]. Moreover, the 2009 project of the Italian Ministry of Health “Smoke-free mothers” establishes the prenatal smoking goal as less than 5% and a relapse prevention goal as less than 50% (on the base of a relapse rate estimate of about 70%) [34]. Our results show that we are close to these goals, but a general increase in tobacco use in males and females, particularly among young people, was also noted in 2009, after a static period lasting 5 years [15, 16]. Consequently, there is a real risk that smoking indicators among pregnant women could worsen.

Quitting and relapsing proportions at different follow-up times show that most change occurred within 6 months. Between 6 and 12 months there was a stabilization or a slow-down in the transitions regarding smoking behaviour. Heavy smokers are less likely to quit smoking and more likely to relapse compared with light smokers as found in other studies [31, 35–39]. Data from the literature indicate that the smoking relapse rate at 12 months postpartum is about 70–80% [40–43]. Our study shows a smoking relapse rate that is lower. In general, the results of this study, in comparison with reported values in other countries, show better smoking indicators at baseline and also lower relapsing rates at follow-up. Moreover, a sensitivity analysis was performed on women who quit during pregnancy. At each follow-up time, all women lost to follow-up were included in the analysis as they were relapsers. The results show estimates of relapsing rates of 26% within 3 months and 67-68% within 6 and 12 months, still lower in comparison with the literature. The strength of this study compared with others is that we have a measure of smoking behaviour collected by reinterviewing mothers at the three follow-up points, a procedure that is likely to reduce biases, in particular recall bias.

The similar prevalences of smoking during pregnancy and at follow-up for breastfeeding women (about 5–7%) suggest that these women are equally aware of the possible harmful effects for fetuses, during pregnancy, and for their children through breastfeeding, although the effects of nicotine on nursing infants are still largely unknown. It has been reported that maternal smoking may reduce the protective effect of breastfeeding (e.g., on respiratory allergy and infections, on Sudden Infant Death Syndrome) [44, 45] but it is not a contraindication to breastfeeding which remains beneficial and appropriate [46–48] and should be promoted also for those women who have difficulty with smoking cessation. However, health care professionals should advise all tobacco-using mothers to avoid smoking within the home and to smoke prior to breastfeeding.

In line with published literature [21, 39, 49, 50], factors associated with smoking behaviour changes during and after pregnancy in our study were education, marital status, parity, smoking intensity, and employment status at follow-up and breastfeeding. We also considered the participation in AC during pregnancy as a predictor factor and found a significant association with a reduction in smoking relapse risk. Breastfeeding, participation in AC, and smoking intensity before pregnancy represent the strongest factors associated with smoking relapse. We found that highly educated women were more likely to quit smoking and less likely to relapse. Participation in AC had no effect on quitting in pregnancy but reduced the smoking relapse risk. This may depend on the late beginning of these courses, which generally start around the 7th month of pregnancy. A possible effect of this factor on quitting smoking could be estimated if they started no more than 3-4 months after conception. Occupational status is also associated with smoking relapse risk. The risks are lower for women who were employed before pregnancy who stopped working during pregnancy and had not yet restarted working at 12 months. This may reflect the fact that women who plan not to return to work and those who do plan to return to work may have different perspectives about breastfeeding and tobacco use. It is also likely that stopping working for the period of maternity reduces work stress conditions that have been described in literature as risk factors for tobacco use [51].

Some studies have focused on the potential association between maternal mental health, such as low mood or stress, and quitting smoking during pregnancy or smoking relapse after delivery. The results are not exactly comparable given the different stress/mood measures used and, in fact, the need for a standard stress scale has been noted [21, 38]. A relationship between postpartum psycological symptoms, breastfeeding and smoking relapse was also hypothesized [21, 27, 52, 53], although the nature of the relationship remains unclear. Our measure of maternal mood is an unspecific measure of distress that must not be confused with a postpartum depression marker. The strength of our study is in the availability of repeated measures of maternal mood, breastfeeding, and smoking behaviour at each follow-up time. Results show a significant independent effect of both maternal mood and breastfeeding on smoking relapse at 3 and 6 months even after adjusting for other potential confounders, while no effect of maternal mood was found at 12 months. This result might reflect the fact that the first months postpartum are a period in which mothers are subjected to a strong systematic source of stress, in particular sleep deprivation or sleep interruption. Previous studies on postpartum relapse and depressive symptoms or stress have reported contradictory results. Some studies found a statistically significant association between maternal mood and smoking relapse within 6 months, but only if breastfeeding was not included as a covariate in the analysis [21, 43]. Others found an independent effect of breastfeeding and stress on smoking relapse, although stress was not statistically significant [18]. Since breastfeeding and maternal mood may be linked [52–54], interaction effects were also considered in post hoc regression analyses, but no significant results were found in our study.

The required information on the previous pregnancy for multiparous women gave us the opportunity of investigating retrospectively the association between breastfeeding a previous infant and smoking behaviour before the following pregnancy. The hypothesis we tested was that a prolonged breastfeeding might produce a reduction in smoking relapse, which is persistent in time. In our study, the values of risk parameters are not in contrast with this hypothesis but still it is not clear which causal mechanism may link breastfeeding with smoking behaviour. We could not determine if women breastfeeding the previous infant for more than 3 months were also less frequently smoking before the previous infant. In order to find some indications, we focused our attention on the present primiparous women assimilating them to the present multiparous women at the time of their previous infant. No statistically significant association was found between smoking behaviour before present pregnancy and breastfeeding at birth or at 3 months from partum, while a borderline but still not significant association was found with breastfeeding at 6 months. These results, even with all constrained assumptions and limits, might support our original hypothesis, which is consistent with the finding of several other studies that breastfeeding may protect against postpartum smoking relapse [17–19, 35, 48].

In conclusion, our population-based study shows that women who are breastfeeding smoke less than not breastfeeding women. A strong association between tobacco use and breastfeeding was found even after controlling for other predictors. A low maternal mood increases the risk of smoking relapse within 6 months. This study also suggests that prolonged breastfeeding reduces the risk of smoking relapse and that this reduction may be persistent in time. Thus, interventions targeting breastfeeding promotion may indirectly support also smoking cessation, even in absence of specific interventions.

## Figures and Tables

**Table 1:** Sample characteristics.

	Completed follow-up		Lost to follow-up		
Variables	sample		sample		*χ* ^2^
	*N* = 2546		*N* = 988		
	*N*	Weighted %	*N*	Weighted %	*P*
*Age *					
<30	727	28.0	369	37.2	0.005
30–34	987	39.6	336	34.6	
>35	832	32.4	283	28.2	
*Education*					
Low	743	27.4	337	34.4	<0.001
Medium	1267	48.8	467	46.2	
High	536	23.8	184	19.4	
*Marital status*					
Married	2015	77.6	738	73.2	0.100
Single/Sep/Div/Wid	531	22.4	250	26.8	
*Employment*					
No	776	28.2	376	36.3	0.003
Yes	1770	71.8	612	63.7	
*Parity*					
Primiparous	1368	55.5	477	49.8	0.036
Multiparous	1144	44.5	483	50.2	
*Multiparous not breastfeeding previous birth or breastfeeding less than 3 months *	*387*	*35.4*	*174*	*39.5*	*0.268*
*Multiparous breastfeeding previous infant more than 3 months *	*754*	*64.6*	*304*	*60.5*	
*Participation in antenatal classes*					
No	1535	59.3	727	76.3	<0.001
Yes	995	40.7	253	23.7	
*Smoking behaviour before pregnancy*					
No	1964	78.4	743	75.2	0.335
Yes	582	21.6	245	24.8	
*Quit during pregnancy *	*397*	*68.7*	*161*	*67.1*	*0.651*
*Smoking during pregnancy *	*183*	*31.3*	*84*	*33.0*	
*Complete breastfeeding at discharge*					
No	*669*	*29.6*	*310*	*32.5*	*0.188*
Yes	*1837*	*70.4*	*659*	*67.5*	

**Table 2:** Smoking behaviour description before, during, and after pregnancy.

No. of cigarettes	Smoking before pregnancy *n*	Quit during pregnancy *N* (weighted %)	Weighted % relapse (% relapse among breastfeeding women)		
			within 3 months	within 6 months	within 12 months
≤5	209	173 (84.7)	11.5 (4.0)	21.2 (6.5)	24.5 (11.8)
6–10	187	127 (65.2)	21.5 (12.4)	33.6 (16.9)	33.8 (25.3)
>10	184	97 (55.2)	26.6 (15.3)	41.5 (23.3)	43.3 (27.7)
Total	580	397 (68.7)	18.5 (9.5)	30.3 (14.3)	32.3 (21.1)

**Table 3:** Logistic regression for smoking behaviour before pregnancy, for quitting smoking during pregnancy and for relapsing within 12 months from partum.

Characteristics	All women *N* = 2546				Smoking before pregnancy *n* = 580				Quitting smoking during pregnancy *n* = 397			
	Smoking before pregnancy *n* = 580				Quitting during pregnancy *n* = 397				Relapse within 12 months *n* = 136			
	Weighted % smoking	OR	95% CI		Weighted % quitting	OR	95% CI		Weighted % relapse	OR	95% CI	
*Mother's age*												
≤30	25.8	***1***			65.9	***1***			38.4	***1***		
>30	19.9	***0.98***	0.78	1.23	70.2	***1.06***	0.60	1.88	29.5	***0.87***	0.41	1.85
*Education*												
low	28.2	***1***			59.5	***1***			38.5	***1***		
medium	20.2	***0.65***	0.48	0.89	72.5	***1.76***	0.93	3.32	35.0	***1.14***	0.55	2.37
high	16.6	***0.50***	0.37	0.67	77.5	***2.42***	0.94	6.27	16.9	***0.60***	0.25	1.45
*Marital status*												
married	17.8	***1***			76.0	***1***			32.2	***1***		
single/sep/div/wid	34.7	***2.30***	1.75	3.02	55.8	***0.38***	0.21	0.71	32.7	***1.00***	0.57	1.75
*Parity*												
primiparous	24.9	***1***			70.0	***1***			29.4	***1***		
multiparous	24.6	***1.06 ***	0.71	1.59	66.2	***0.61 ***	0.39	0.96	38.4	***1.28 ***	0.55	2.96
*Antenatal classes*												
no					66.8	***1***			42.3	***1***		
yes					71.9	***0.97***	0.57	1.62	18.9	***0.43***	0.19	0.96
*Kind of smoker*												
light					75.4	***1***			28.4	***1***		
heavy					55.2	***0.45***	0.31	0.64	43.3	***2.38***	1.44	3.91
*Any Breastfeeding *												
no									37.6	***1***		
yes									21.5	***0.43***	0.19	0.94
*Employment *												
no	23.5	***1***			62.8	***1***			52.5	***1***		
yes-restart working	20.8	***1.00***	0.78	1.26	71.4	***1.12***	0.57	2.20	28.7	***0.47***	0.21	1.06
yes-not restart working									21.8	***0.36 ***	0.09	1.36

**Table 4:** Smoking relapse and the association with breastfeeding and maternal mood at each follow-up.

Characteristics	Follow-up 3 months*				Follow-up 6 months*				Follow-up 12 months*			
	*n* = 461				*n* = 406				*n* = 415			
	Weighted % relapse	OR**	95% CI		Weighted % relapse	OR**	95% CI		Weighted % relapse	OR**	95% CI	
Breastfeeding***												
No	29.3	1			47.0	1			35.2	1		
Yes	8.4	0.23	0.09	0.58	14.3	0.22	0.11	0.44	19.6	0.41	0.18	0.92
*Maternal mood*												
High	16.8	1			28.3	1			34.6	1		
Low	25.0	1.73	1.21	2.47	41.9	1.73	1.03	2.90	26.9	0.71	0.46	1.10

*Based on samples interviewed at each follow-up time.

**OR adjusted by all the variables indicated in Table 3.

***Breastfeeding: “full” at 3 months; “any” at 6 and 12 months.
